# A Case of Bordetella bronchiseptica Bacteremia in a Patient With COVID-19: Brief Report

**DOI:** 10.7759/cureus.15976

**Published:** 2021-06-27

**Authors:** Styliani Papantoniou, Antonios Tsakiris, Theodoros Ladopoulos, Georgios Kranidiotis, Charalampos Tamvakos

**Affiliations:** 1 Department of Internal Medicine and Diabetes Center, Tzaneio General Hospital of Piraeus, Piraeus, GRC; 2 Department of Neurology, St. Josef Hospital, Ruhr University Bochum, Bochum, DEU

**Keywords:** covid, bacteremia, neutrophils-lymphocytes-ratio, opportunistic, dexamethasone, bordetella bronchiseptica

## Abstract

Bordetella bronchiseptica is a gram-negative coccobacillus that colonizes the respiratory system of mammals such as dogs, cats, rabbits and others and might cause upper respiratory tract infections. Although it can be rarely pathogenic in humans, there are several case reports describing infections in humans. We describe the case of a patient without prolonged immunosuppression or underlying diseases, with bacteremia from Bordetella bronchiseptica, while being treated in a tertiary hospital for COVID-19 infection.

## Introduction

Bordetella bronchiseptica can rarely cause infection in humans [1]. However, various case reports describe infections such as pneumonia, bacteremia, and pancreatic abscess in patients with immunocompromising conditions [2]. In the literature, there are reports of Bordetella bronchiseptica co-infection in patients with COVID-19, mostly related to respiratory infections [1]. We describe the first case of bacteremia in an immunocompetent patient with no underlying comorbidities who was hospitalized due to severe COVID-19.

## Case presentation

A 47-year-old patient presented to the emergency department of a tertiary hospital in March 2021 with fever up to 39°C which started six days prior to admission. The patient did not report any health problems or any home medications. A molecular test was performed by PCR for SARS-CoV-2, which was positive. Clinical examination revealed dryness of the mucous membranes and diffuse bilateral crackles. No other pathological findings were found during the clinical examination. Notable findings in the laboratory tests were leukocytosis (WBC 32,190/mm^3^), neutrophilia (Neu 31,200/mm^3^), and severe lymphopenia (Lymph 470/mm^3^). The patient’s virological test was negative for HIV, hepatitis C virus (HCV), and hepatitis B virus (HBV). Treatment of the patient upon admission included antiviral therapy with remdesivir, intravenous dexamethasone at a dose of 6 mg due to respiratory failure, prophylactic anticoagulant therapy with low molecular weight heparin, and empirical antibiotic therapy with ceftriaxone and azithromycin. On the second day of hospitalization, his respiratory status deteriorated and his treatment was escalated in a high-flow nasal cannula. The patient’s respiratory condition de-compensated requiring endotracheal intubation and transfer to ICU. Our patient on the 10th day of his hospitalization in the Intensive Care Unit developed a new fever of 39.6°C. Bordetella bronchiseptica, Candida tropicalis and Enterococcus faecium were isolated in blood cultures obtained from the central venous line, which was inserted before admission to ICU, while only Bordetella bronchiseptica was isolated peripherally (Table 1).

**Table 1:** Blood Culture Results bbr: Bordetella bronchiseptica; MIC: Minimal Inhibitory Concentration; S: Susceptible; IE: Insufficient evidence to set a clinical breakpoint; R: Resistant; CRB: Catheter-related bacteremia.

	bbr	
Antimicrobial	MIC	Result
Amikacin	<=2	S
Cefotaxime	8	R
Ceftazidime	4	IE
Gentamicin	<=1	S
Imipenem	<=0.25	S
Meropenem	<=0.25	S
Tigecycline	<=0.5	S
Differential time to positivity	Negative for CRB	

Central venous catheter was replaced and antibiotic treatment was escalated to piperacillin / tazobactam, linezolid, and caspofungin. The patient remained hemodynamically stable, was successfully extubated on the 14th day of his ICU hospitalization and he returned to the COVID-19 clinic to continue his treatment. Three days later and while our patient was receiving the antibiotic treatment, he presented a new fever and new blood cultures were obtained. Bordetella bronchiseptica was isolated in both the blood culture sets taken from the central venous line and the set taken from the peripheral venous line. The strain isolated was the same as the one isolated before. Antibiotic treatment was escalated to meropenem, while linezolid and caspofungin were discontinued. The patient remained clinically and hemodynamically stable and received treatment for Bordetella bronchiseptica bacteremia for a total of 14 days. He was discharged after 24 days after his admission. At the follow-up of the patient one month after discharge, the patient did not present any clinical complications and the laboratory parameters had recovered to normal levels.

## Discussion

Bordetella bronchiseptica is a gram-negative coccobacillus that colonizes the respiratory system of mammals, mainly dogs, cats and hares and causes infections of the respiratory system [3]. Although it can be rarely pathogenic in humans, several case reports are describing various infections such as pneumonia, bacteremia, pancreatic abscess in patients with immunocompromising conditions [2], while there are reports of Bordetella bronchiseptica pneumonia in patients with COVID-19 [1]. The microorganism settles inside the epithelial cells and escapes the host's innate immune response. This allows it to remain in the host's respiratory system for a longer period, releasing toxic products and bacteria. In the case of patients with immunosuppression, this procedure can be further accelerated, as the compromised mechanisms of the innate immune system make it even more difficult to treat the microorganism [3].

According to a recent meta-analysis, the incidence of bacterial infections in patients with COVID-19 is 6.9% and is significantly lower compared to that reported in patients with influenza [4]. Bacterial infections are more common in patients with severe disease and occur later in viral infection. The pathogenetic mechanism has not been clarified. In patients with COVID-19, gram-negative pathogens are the leading cause of bacterial infections. The pathogenic microorganism responsible for bacteremia usually originates from the microbiota of the respiratory system, especially in immunocompromised patients. Prolonged invasive mechanical ventilation appears to play an important role in the development of a secondary bacterial infection. The destruction of airway barriers due to ventilator-associated injury in combination with the immune-related epithelial damage caused by SARS-CoV-2 leads to the invasion of opportunistic bacteria which have colonized the respiratory system [4].

Our patient presented an increased neutrophils-lymphocytes-ratio (NLR), a marker that indicates a disturbed immune response and has been associated with a worse outcome in patients with COVID-19. The role of neutrophils in the mechanisms of innate immunity is of vital importance, while lymphocytes are essential for the inflammatory response of the patient. NLR reflects the body's response to the infection, as an increase shows neutrophil rising, lymphocyte apoptosis, or both. Studies have shown that NLR has a higher prognostic value compared with other inflammatory markers for the diagnosis of bacteremia [5]. Although the NLR can be influenced by patient's comorbidities and could be a better criterion for the rational administration of antibiotics to patients with COVID-19 [6].

Despite we did not fully evaluate the immune sufficiency of our patient, we consider the patient as immunocompetent. Our patient was HIV negative, did not report underlying diseases or medication while his medical history did not reveal recurrent infections or opportunistic infections. Besides follow-up, laboratory parameters, including the number of patient white blood cell populations, returned to normal levels. In a study that aimed to develop an algorithm for the detection of immunocompromised patients with severe sepsis, patients were classified based on chart review as having or not an immunosuppressive condition. This algorithm detected immunocompromised patients in over 90% [7]. Our patient received treatment with dexamethasone to prevent COVID-19 immunopathology. According to existing data, it does not seem that dexamethasone at this dose and duration of treatment associated with immune complications [8].

The overuse of broad-spectrum antibiotics is a potential key factor in the development of secondary bacterial infections in patients with COVID-19, as it alters the gut and pulmonary microbiome and disrupts the gut-lung axis. This reduces the bacterial populations that are beneficial for the host's innate immunity and limits the action of the gut microbiome in enhancing host immunity [9]. In this way, it creates an environment that favors the growth of resistant microorganisms to these antibiotics, which in combination with the immune dysregulation of patients with COVID-19, can lead to bacterial superinfection [10]. Bordetella bronchiseptica has reduced susceptibility to cephalosporins, as low membrane permeability of the pathogen prevents antibiotics from entering intact cells [11]. Although Bordetella bronchiseptica is usually susceptible to piperacillin / tazobactam, genes such as BOR-1 have been identified as responsible for hydrolysis of B-lactams [12]. In previous case reports, azithromycin has been used effectively to treat respiratory infections by Bordetella bronchiseptica [1]. The failure of treatment with azithromycin in our patient is probably due to the pharmacokinetic properties of the antibiotic, as the concentrations of azithromycin in tissues such as the lung, are much higher than those in plasma [13].

## Conclusions

We reported a case of bacteremia from Bordetella bronchiseptica in a patient being treated for COVID-19. Although the incidence of bacterial infections is relatively low, dysregulation of the immune response and complications caused by SARS-CoV-2 in combination with overuse of broad-spectrum antibiotics can lead to secondary bacterial infections from opportunistic pathogens even in immunocompetent patients. Although there is no specific laboratory sign, an increased neutrophils-lymphocytes-ratio can lead to a high index of suspicion for coinfection among COVID-19 patients. The intrinsic resistance of Bordetella bronchiseptica to B-lactams and the pharmacokinetics of azithromycin complicated the patient's clinical course.
